# Regeneration of the dermal skeleton and wound epidermis formation depend on BMP signaling in the caudal fin of platyfish

**DOI:** 10.3389/fcell.2023.1134451

**Published:** 2023-02-09

**Authors:** Lana Rees, Désirée König, Anna Jaźwińska

**Affiliations:** Department of Biology, University of Fribourg, Fribourg, Switzerland

**Keywords:** *Xiphophorus maculatus*, neoteleosts, lepidotrichia, actinotrichia, basal epithelium, actinodin, TP63

## Abstract

Fin regeneration has been extensively studied in zebrafish, a genetic model organism. Little is known about regulators of this process in distant fish taxa, such as the *Poeciliidae* family, represented by the platyfish. Here, we used this species to investigate the plasticity of ray branching morphogenesis following either straight amputation or excision of ray triplets. This approach revealed that ray branching can be conditionally shifted to a more distal position, suggesting non-autonomous regulation of bone patterning. To gain molecular insights into regeneration of fin-specific dermal skeleton elements, actinotrichia and lepidotrichia, we localized expression of the *actinodin* genes and *bmp2* in the regenerative outgrowth. Blocking of the BMP type-I receptor suppressed phospho-Smad1/5 immunoreactivity, and impaired fin regeneration after blastema formation. The resulting phenotype was characterized by the absence of bone and actinotrichia restoration. In addition, the wound epidermis displayed extensive thickening. This malformation was associated with expanded Tp63 expression from the basal epithelium towards more superficial layers, suggesting abnormal tissue differentiation. Our data add to the increasing evidence for the integrative role of BMP signaling in epidermal and skeletal tissue formation during fin regeneration. This expands our understanding of common mechanisms guiding appendage restoration in diverse clades of teleosts.

## Introduction

Fin regeneration has been documented in many teleost species, since the first report of this phenomenon in goldfish more than two hundred years ago (Broussonet, 1786). Among all fins, the caudal appendage has been recognized as particularly suitable for regenerative studies, as it is easily accessible for surgery, imaging and morphometric analysis (Akimenko et al., 2003). A typical teleost caudal fin has a dorso-ventrally symmetrical fanlike shape with a narrower base and an expanded distal margin, which can be forked, lunate, truncate or rounded in different fishes (Flammang, 2014). The molecular studies of fin regeneration have been mostly conducted in leading model species, namely the zebrafish and the medaka, which are amenable to genetic approaches (Katogi et al., 2004; Kirchmaier et al., 2015; Marques et al., 2019; Sehring and Weidinger, 2020). Whether the knowledge gained from these fishes can be generalized for distinct clades of teleosts remains unclear, as few comparative studies with non-model fishes have been performed. The zebrafish belongs to the *Cypriniformes* order, which is placed at a basal phylogenetic position in the *Otophysa* section and among freshwater bony fishes (Nakatani et al., 2011; Betancur-R et al., 2017) (Supplementary Figure S1). Little is known about regenerative mechanisms in phylogenetically more recent orders of *Neoteleostei*, such as *Cyprinodontiformes*.

Anatomical studies of various extant and extinct fishes have revealed that the main part of the caudal fin derives from the tissue below the notochord/vertebral column (Goodrich, 1930; Metscher and Ahlberg, 2001; Hadzhiev et al., 2007; Schultze and Arratia, 2013; Arratia, 2015; Desvignes et al., 2018). Evolutionary analysis has suggested that “the teleost caudal fin is actually the ventral lobe of the ancestral fin” (Sallan, 2016). Thus, despite its external dorsoventral symmetry along the midline of the body, the caudal fin can be considered as a ventral appendage. Although this bauplan is highly conserved among teleosts, including the zebrafish and the medaka, we have recently identified a rule-breaking variation in the platyfish (*Xiphophorus maculatus*), a member of the Poeciliidae family of the *Cyprinodontiformes* order (Rees et al., 2022). This taxon belongs to the *Ovalentaria* series in neoteleosts, which are considered as modern teleosts, compared to the group represented by zebrafish (Betancur-R et al., 2017) (Supplementary Figure S1). Diverging from the typical anatomical rules of teleosts, the platyfish caudal fin includes principal rays originating from mesenchyme above the notochord, leading to an unconventional dorso-ventral “hybrid” fin (Rees et al., 2022). This finding provides evidence of an unexpected diversity in appendage development among teleosts. Inspired by the innovative caudal endoskeleton in the platyfish, we set out to investigate the molecular bases of dermal skeleton regeneration in this species.

**FIGURE 1:**
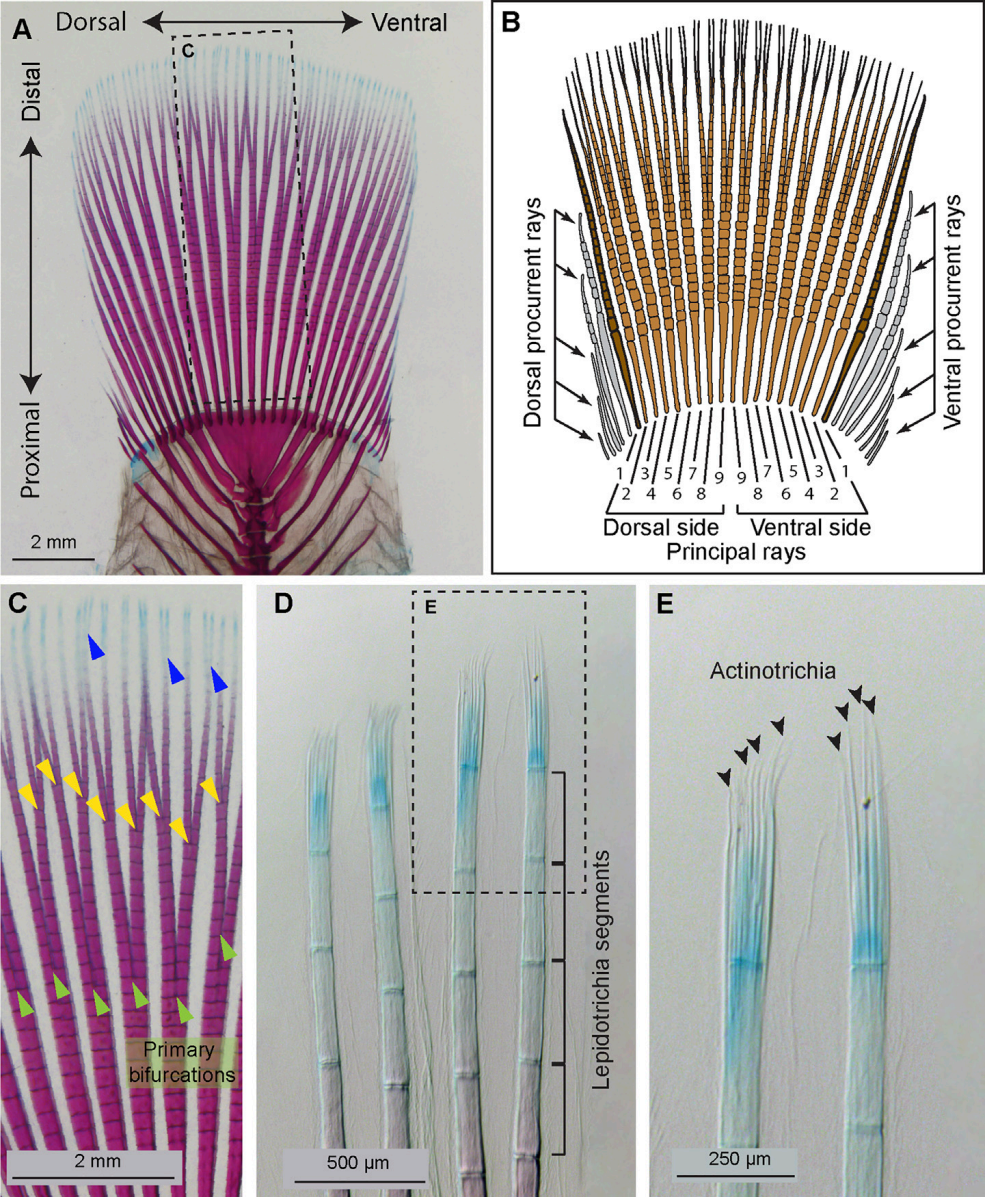
Anatomy of the platyfish caudal fin. **(A and C–E)** A caudal skeleton of adult platyfish, stained with alcian blue and alizarin red to detect cartilage and mineralized bone, respectively. **(B)** A schematic of the platyfish caudal fin illustrates a compound organization of the appendage. The distal margin of the fin is supported by typically 18 principal rays, among which 16 are branched (light brown) and two unbranched (dark brown). The base of the fin is widened by procurrent rays (grey) that are short and unbranched. **(C)** A magnification of the framed area in **(A)**, with indicated ray branchpoints: primary (green arrowheads), secondary (yellow arrowheads), and occasional tertiary (blue arrowheads) ray bifurcations. **(D)** The distal tip of rays comprises non-calcified lepidotrichia leading to a gradient of alizarin red and alcian blue staining along the proximal-distal axis. Segmentation of the rays is visible at this magnification. **(E)** A higher magnification of the framed area in **(D)** allows visualization of unstained actinotrichia filaments (black arrowheads) through contrast imaging. Actinotrichia are located at the level of the distal-most segment of the rays.

In contrast to the tetrapod limb, the caudal fin is a compound organ consisting of a sequence of rays, which are autonomous units. The main rays, called principal rays, extend from the base to the distal margin, whereas peripheral procurrent rays are shorter, ending laterally before reaching the distal fringe. The platyfish caudal fin has a rounded shape supported by approximately 18 principal rays (Figures 1A, B). Among them, 16 rays are branched plus one unbranched at the dorsal and the ventral side, as defined by anatomical conventions (Schultze and Arratia, 1989; Arratia, 2008; Schultze and Arratia, 2013; Arratia, 2015). Ray branches, also called bifurcations, are formed through dichotomous splitting of the source ray during their distal growth, which occurs at the tip of the fin. Ray bifurcations are induced progressively during the lifespan, whereby the primary branches are proximal and the most recent branches are distal (Figure 1C). Adult fish can have one, two, three and occasionally four ramification levels of their principal rays, depending on factors that remain unknown. While the primary bifurcations are usually at a similar proximo-distal position among individual fish of the same strain, the subsequent bifurcations are usually unpredictably variable. Thus, the primary ray bifurcation can be considered as the most informative landmark of fin proximo-distal patterning.

The regulation of ray branching has been addressed in zebrafish, which has a forked margin of the caudal fin (Johnson and Bennett, 1999; Tu and Johnson, 2011). During fin regeneration, ray splitting requires Sonic hedgehog (Shh) signaling between the basal epithelium and underlying dedifferentiated osteoblasts (Laforest et al., 1998; Quint et al., 2002; Hadzhiev et al., 2007; Zhang et al., 2012; Armstrong et al., 2017; Braunstein et al., 2021). Beside this permissive molecular signal, the inductive mechanisms of ray branching seem to be complex. Although the regenerated fin recapitulates its initial form, the primary ray bifurcations are distally shifted relative to the original developmental position (Akimenko et al., 2003; Azevedo et al., 2011). Furthermore, ray branching might be further distally displaced or even suppressed through a physical separation of adjacent rays during regeneration (Marí-Beffa et al., 1999; Murciano et al., 2002; Dagenais et al., 2021). Another interesting observation is that the newly formed twin rays can be gradually stitched together, depending on the balance between bone mineralization and resorption during ray branching formation (Cardeira-da-Silva et al., 2022). Thus, ray branching is not robustly recapitulated during regeneration, but it is subjected to modulation by non-autonomous factors, such as osteolytic tubules, surface geometry and interaction with adjacent tissue. Whether regeneration of ray branchpoints is faithfully reproduced in the rounded fin of platyfish, and whether this process is susceptible to non-autonomous inputs remains unknown.

The caudal fin contains a dermal skeleton, also called the exoskeleton, which is located between the epidermis and mesenchyme. Each ray contains a pair of parentheses-shaped hemirays, called lepidotrichia, which are synthesized by osteoblasts, positioned below the epidermis (Akimenko et al., 2003; Pfefferli and Jaźwińska, 2015; Sehring and Weidinger, 2020). Mature lepidotrichia are mineralized bones, metamerically interspaced by joints that subdivide the rays into segmental units (Figures 1B, D). The tip segment is located within the growth zone of the fin, and it contains undifferentiated osteoblasts and unmineralized bone (Knopf et al., 2011; Sehring et al., 2022). This immature tissue is supported by actinotrichia, which are fin-specific skeletal elements (Zhang et al., 2010). Actinotrichia are non-mineralized spicules, organized into a brush-like structure at the tip of each ray, as shown in the platyfish fin (Figure 1E). Like lepidotrichia, actinotrichia occupy the epidermal-mesenchymal interface, whereas the interior space of the ray is filled with vascularized and innervated mesenchymal tissue (Mari-Beffa and Murciano, 2010; Pfefferli and Jaźwińska, 2015; Sehring and Weidinger, 2020). Actinotrichia consist of extracellular structural proteins, mainly collagens and actinodins, displaying a regular cross-banding visible with electron microscopy (Witten and Huysseune, 2007; Huang et al., 2009; Duran et al., 2011; König et al., 2018). Little is known about the expression of *actinodin* genes and the molecular regulation of actinotrichia regeneration in the recent orders of neoteleosts, represented by the platyfish.

After fin amputation, the wound epithelium covers the injured tissue, which is a necessary process for dedifferentiation and proliferation of mesenchymal cells and osteoblasts of the stump (Mari-Beffa and Murciano, 2010; Pfefferli and Jaźwińska, 2015; Sehring and Weidinger, 2020). These activated cells migrate distally and form a blastema, which comprises precursor cells of the new tissue. Early blastema formation is dependent on FGF signaling in zebrafish and platyfish (Whitehead et al., 2005; Offen et al., 2008). The regenerative outgrowth is initially supported by actinotrichia prior to lepidotrichia formation. Actinotrichia provide mechanical support to the undifferentiated ray tips, and they may act as a scaffold for distally migrating blastema cells (Wood and Thorogood, 1984; Zhang et al., 2010; Duran et al., 2011; van den Boogaart et al., 2012; Bhadra and Iovine, 2015). In zebrafish, regeneration of the dermal skeleton is dependent on Bone morphogenetic protein (BMP) signaling (Quint et al., 2002; Stewart et al., 2014; Thorimbert et al., 2015; Wehner and Weidinger, 2015). Inhibition of this pathway impairs lepidotrichia and actinotrichia formation, resulting in blocked fin regeneration. Furthermore, thickening of the epidermis has been reported, but the significance of this phenotype remains undefined. Whether the BMP-dependent mechanism of wound epidermis and dermal skeleton differentiation is conserved in phylogenetically modern orders of neoteleosts remains unknown.

Here, we characterized fin regeneration in adult platyfish, focusing on the role of BMP signaling during this process. Firstly, we investigated the restoration of the primary ray bifurcation, which is the earliest ontogenetic landmark of the proximo-distal patterning of the fin skeleton. To understand the molecular origin of actinotrichia regeneration, we investigated the expression of four *actinodin* orthologs in the platyfish blastema. To identify the contribution of Actinodin-1, we generated a platyfish-specific antibody against this protein, and we analyzed its distribution in the blastema. For functional studies, we applied a pharmacological approach to inhibit BMP signaling. Based on phospho-Smad1/5 immunostaining, the efficacy of the inhibition was validated. To understand the role of BMP activity in the fin, we analyzed cell proliferation, differentiation of the wound epidermis and the deposition of extracellular matrix components of the blastema and dermal skeleton. Our analysis revealed that BMP regulates the formation of wound epidermis, lepidotrichia and actinotrichia, which are essential for progression of regeneration in the platyfish caudal fin.

## Materials and methods

### Animal procedures and drug treatments

Platyfish (*X. maculatus*) were females and males of the “Mickey Mouse” strain, at approximately 3-4 cm standard length. They were purchased from a commercial fish vendor (Aqualand, Renens/Lausanne, Switzerland). During fin regeneration without treatments, the fish were maintained in tanks at room temperature. Animal housing and experimentation was approved by the Cantonal Veterinary office of Fribourg, Switzerland.

For fin amputation, the fish were immersed in analgesic solution of 5 mg/L lidocaine for 1 h before the procedure. Then, fish were anesthetized in 0.6 mM tricaine (MS-222, ethyl-m-aminobenzoate, Sigma-Aldrich). Fin amputations were performed with a sharp blade. The position of the amputation plane was at a distance of approximately three ray segments proximally to the primary ray bifurcation. Bright field images of fins were taken with a Leica AF M205 FA stereomicroscope.

Before fin collection for fixation, fish were euthanized by immersion in 0.6 mM tricaine solution for at least 10 min. The fins were cut off with a sharp blade and fixed in 4% paraformaldehyde (PFA, Sigma-Aldrich).

Drug treatments were performed in an incubator at 27°C. LDN193189 (HY-12071, MedChemExpress) was dissolved in acidified DMSO to achieve 10 mM stock solution, which was then aliquoted and stored at −20°C. The final concentration of the drug was 10 μM diluted in the total volume of 400 mL fish water. The final drug solution was adjusted to pH 7.4, like the control. Control fish were kept in 0.1% DMSO, which corresponds to the solvent concentration of the LDN193189 treated fish. For cell proliferation analysis, 100 mg BrdU (B5002, Sigma-Aldrich) was dissolved in 20 ml distilled water at 37°C to achieve the stock concentration of 5 mg/ml, aliquoted, and stored at −20°C. For treatment, these aliquots were used diluted 1:100 in the drug or DMSO-containing fish water to a final concentration of 50 mg/L BrdU.

### Alcian blue and alizarin red staining of whole fins

Fins were double stained for mineralized and non-mineralized bone or cartilage, according to Taylor and Van Dyke (Taylor and Dyke, 1985). Briefly, fins were fixed overnight in 50 ml 4% PF buffered with 1.25 g CaCO_3_. They were rinsed in water, dehydrated in ethanol series, and treated with xylene for 15 min to degrease the specimens. The first staining was conducted in freshly prepared 0.1% alcian blue dissolved in a mix of 70% ethanol and 30% acetic acid for 6–12 h. The specimens were incubated for 1 day in 0.5% KOH to achieve neutralization. Next, bleaching solution was prepared by mixing 1 unit of 0.3% hydrogen peroxide with 9 units of 0.5% KOH. Fins were treated in this bleaching solution for 30 min to remove pigmentation. Clearing of the tissue was performed for several hours in 0.1% trypsin dissolved in a solution consisting of three parts saturated sodium borate and seven parts distilled water. The second staining solution was 0.1% Alizarin red S in a 0.5% KOH solution. Cleaning was performed in distilled water, and scales were removed from the skin. Washing was performed in several rounds of 0.5% KOH, 30 min each until no color was diffusing from the specimens. The fins were transferred through glycerol/0.5% KOH gradient solutions (1:2; 1:1, 2:1), and stored in 100% glycerin.

### *In-situ* hybridization

The genome of platyfish has been sequenced (Schartl et al., 2013), however, its annotation is still incomplete. To identify the *actinodin* and *bmp2* genes in the southern platyfish (taxid:8083), the zebrafish FASTA sequences of the corresponding proteins were entered as query in Standard Protein BLAST in the NCBI website. The highest identity score was used as a parameter to select the closest ortholog. To generate antisense probes, portions of the coding sequences were cloned by the PCR amplification using platyfish cDNA generated from embryos. The reverse primers were synthesized with an addition of the promoter for T3 polymerase. The following forward (Fw) and reverse (Rev) primers were used:

*and1* (XM_023326727.1) Fw 5′-ACG​CTG​TTT​GGA​TCA​CTT​CC-3′; Rev: 5′- AAG​ATC​CAG​GAC​CAG​TGT​GG-3′

*and2* (XM_023335108.1) Fw 5′-GAT​TCC​GAT​GAC​CCA​GAA​TG-3′; Rev: 5′-TGC​ACA​TAG​CAG​TCG​TAG​CC-3′

and3 (XM_023336171.1) Fw 5′-ACA​GCC​TGA​TGG​AAA​TCC​TG-3′; Rev: 5′- CTG​GCT​GAG​CAT​CAC​GAT​AG-3′

*and4* (XM_023328200.1) Fw 5′-GTC​TCA​CTC​CAG​ACC​GAA​GC-3′; Rev: 5′-GCG​GGT​CAT​ACA​GTT​CAT​CC-3′

*bmp2* (XM_005805630.2) Fw 5′-CCG​CTC​TCT​CAT​GGT​ACT​GC-3′; Rev: 5′-TGC​ACG​TCG​CTG​AAA​TCT​AC-3′

Digoxigenin-labeled RNA antisense probes were synthesized from PCR products with the Dig labeling system (Roche). For the detection of transcripts, we followed our previously published protocol (König et al., 2018).

### Production of And-1 peptide antibodies

A peptide EQDDHRAYADDYRKK, which corresponds to the real C-terminus of Actinodin 1 protein in platyfish (XP_023182495.1) with an additional cysteine residue at the amino terminus of the peptide was synthesized, coupled with KLH carrier, and injected into rabbits to generate polyclonal antibodies (Eurogentec). The serum was affinity purified against the peptide and validated using ELISA.

### Immunofluorescence staining on sections

Immunofluorescence analyses of fin cryosections were performed according to our previously published protocol (König et al., 2018). Briefly, fins were harvested, fixed in 4% paraformaldehyde (PFA) overnight at 4°C, equilibrated in 30% sucrose solution, mounted in tissue freezing media (Tissue-Tek O.C.T.; Sakura), cryosectioned at 20 μm thickness using a Hyrax C50 cryostat, collected on Superfrost Plus slides (Fisher Scientific). The following primary antibodies were used: mouse anti-Chondroitin sulfate at 1:500 (C8035, Sigma-Aldrich); rabbit anti-And1 at 1:2000 (this study); rabbit anti-phospho-Smad1/5 (41D10) at 1:100 (#9516, Cell Signaling Technology, Danvers, MA, United States); rabbit anti-tenascin C, 1:500 (T2550-23 USBiological, Hamburg, Germany); rabbit anti-Tp63 at 1:500 (GTX124660, GeneTex, Irvine, CA, United States) rat anti-BrdU at 1:100 (ab6326, Abcam Inc., Cambridge, MA, United States). Fluorescent dye-coupled secondary antibodies (Jackson ImmunoResearch Laboratories, West Grove, PA, United States) were used at 1:500. DAPI (Sigma-Aldrich) was used to label nuclei.

### Whole mount immunofluorescence staining

The staining procedure was adapted from (Wiley et al., 2015).The regenerates were fixed flat in 4% paraformaldehyde at 4°C, then washed in PBS and bleached with 3% H_2_O_2_ during 30 min. They were next rinsed in methanol prechilled at −20°C and incubated overnight in 100% methanol at −20°C. The following day, fins were washed in prechilled acetone at −20°C for 7 min, then rinsed three times in PBST. Then, two digestions were performed: firstly, clearing took place in 0.5% trypsin in PBST for 45 min at 37°C, and an incubation in 0.5% hyaluronidase in PBST at 37°C for 1 h. Both steps were followed by several washes in PBST. Blocking was performed for 1 h in a solution of 5% goat serum and 1% DMSO in PBST. This solution was also used for incubation in the Collagen II antibody at 1:250 (II-II6B3, DSHB, University of Iowa, United States) overnight at 4°C with mild agitation. The following day, the specimens were washed three times in PBST for 10 min each, and re-blocked for 30 min. Finally, the secondary antibody at 1:500 in blocking solution was used overnight at 4°C, with mild agitation. Fins were washed in PBST for 1 h and mounted in 90% glycerol in 20 mM Tris pH 8 with 0.5% N-propyl gallate on glass slides.

### Image analysis and statistics

The specimens were analyzed by confocal microscopy (Leica TCS-SPII) or brightfield color images were taken with a Leica AF M205 FA stereomicroscope equipped with a digital camera (Leica Microsystems, Wetzlar, Germany). All images were processed using ImageJ (NIH) and Photoshop (Adobe). For quantitative analysis of cryosections, at least three images per fin were analyzed.

The lengths of rays were measured in ImageJ by tracing lines from the arch-shaped *arteria* and *vena flabellaria* at the fin base to the tip of the fin or to the bifurcation point. Each fin was represented by an average of the same rays (dorsal rays 6, 7 and 8).

Tp63 expression was analyzed using confocal images of longitudinal sections by drawing 10 straight lines at regular intervals from the basal wound epidermis to the outer epidermis after the plane of amputation. The ImageJ colocalization tool was used to identify dual DAPI+\Tp63+ nuclei. All DAPI+ and dual DAPI+\Tp63+ nuclei that intersected the drawn lines were counted, then an average of these ten measurements was calculated per image. Three images were quantified to create an average per fin.

The BrdU assay was analyzed using the ITCN and colocalization plugins in ImageJ to count all DAPI + and dual DAPI+\BrdU+ nuclei in the blastema and wound epidermis above the amputation plane.

Graph were produced in Graphpad, and error bars correspond to SEM. Statistical analyses were performed using the appropriate Student’s t-tests, as indicated in the figure legends.

## Results

### A distal shift of ray branching is modulated by non-autonomous factors from the adjacent tissues

**FIGURE 2:**
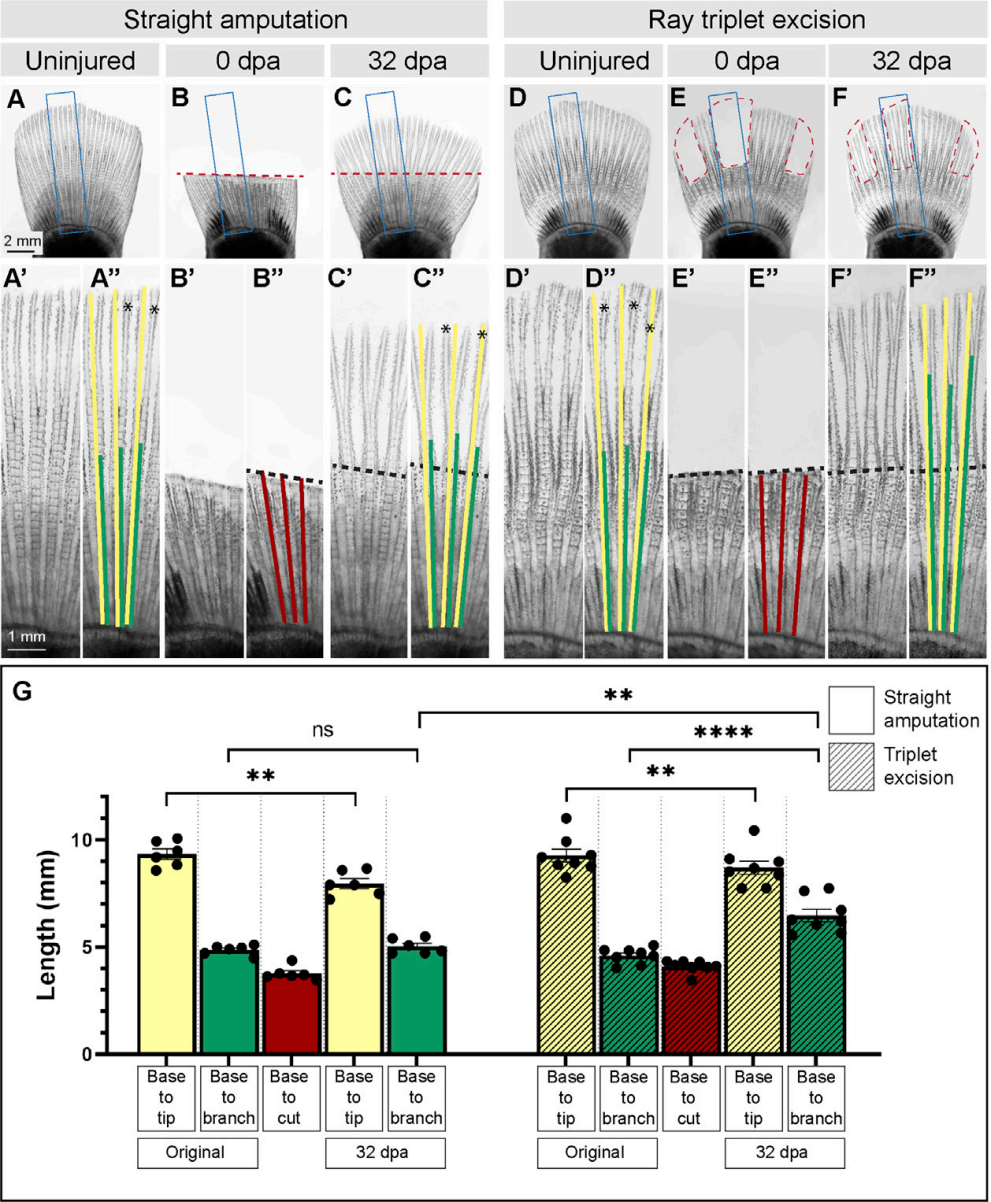
Morphometric analysis of rays and their primary branchpoints in regenerated fins following removal of all or a few rays. **(A–F)** Live imaging of platyfish fins before amputation (uninjured), immediately after amputation (0 dpa) and during the terminal phase of regeneration (32 dpa), in two experiments that involve straight amputation or ray triplet excision, as labeled above the photographs. Blue frames encompass the area that is magnified below the photo, and are labeled with the same letter with a prime symbol (A″, B″, C″, E″, F″). Red dashed lines depict the surgery plane. The colored lines overlap with parts of rays that were measured. Yellow lines correspond to the ray length from base to tip, the green lines depict the length from base to bifurcation point, the red lines demarcate the length from base to the cut. These parameters are plotted in the graph **(G)** for each amputation type. Black dashed lines indicate amputation planes, and asterisks shows secondary bifurcations, if present. Statistics: For straight amputation, N = 6; for ray triplet excision, N = 8; measurements per fin represent average of three rays per fish; error bar, SEM; n.s., not significant, *p* > 0.05; **p < 0.01; ****p < 0.0001; paired two-tailed Student’s t-test within each experiment; unpaired two-tailed Student’s t-test between experiments.

In the regenerating caudal fin of zebrafish, the first ray branchpoint is typically shifted toward a more distal position in comparison to its original location in the intact organ (Azevedo et al., 2011). To determine whether a similar morphogenetic phenomenon occurs in platyfish, we amputated fins along a straight plane proximally to ray branching (Figure 2A, B″). At 32 dpa, the fins regenerated their rounded shape, and the rays restored the first bifurcation (Figure 2C-C″). To precisely compare the ray length and the bifurcation position in the original fin and its regenerated copy, we sampled the specimens by selecting three principal rays that correspond to rays 6, 7 and 8, counted from the dorsal side (Figure 1B; Figures 2A′-C″). The ray base was defined as the arch-shaped *arteria* and *vena flabellaria*, which are stem blood vessels of the fin vasculature (Schultze and Arratia, 2013). In the intact fins, the length of the selected rays was approximately 9 mm, whereas the distance between the base to the bifurcation point was nearly 5 mm (Figures 2A-A″, G). To investigate regeneration of the ray branching points, the fin was cut 1 mm proximally to this structure (Figures 2B-B″, G). At 32 dpa at room temperature, the total ray length was 8 mm, approaching the termination stage of regeneration. Interestingly, we found that the ray branching point occurred at the distance of 5 mm, which is the same position as in the original fin (Figures 2C-C″, G). We concluded that the distal shift of the first ray bifurcation point, which was described in the zebrafish, does not occur in the platyfish. Thus, the original pattern of primary ray branchpoints is faithfully recapitulated in regenerated platyfish fins.

Upon amputation, each cut ray establishes its own autonomous blastema that acts as an independent signaling center during regeneration (Pfefferli and Jaźwińska, 2015; Sehring and Weidinger, 2020). Ray branching can be modulated by narrowing the fin width or by surgical separation of adjacent rays during regeneration, as shown in zebrafish (Murciano et al., 2002; Dagenais et al., 2021). To determine whether a similar plasticity in the specification of ray branching can be experimentally induced in platyfish, we amputated three sets of three rays interspaced by intact tissue. Specifically, we excised the principal rays 1/2/3 and rays 6/7/8 of the dorsal fin half, and the rays 1/2/3, counted from the ventral margin (Figures 1B, 2D, E). The resulting uneven shape of the fin is predicted to modulate hydrodynamic forces on the appendage, and to allow a lateral interaction between the intact pre-existing tissue and newly forming outgrowth (Dagenais et al., 2021). To compare the results between straight amputation and ray excision experiments, we used animals with similar fin sizes (Figures 2A, D, G). At 32 days after ray excision, the missing tissue was regenerated (Figure 2F-F″). Like in the previous experiment, the length of rays was slightly shorter, compared to the original size, approaching the terminal phase of regeneration (Figures 2F-F″, G). Importantly, the distance between the base to the branching position reached approximately 6 mm, which is significantly higher than in the original fin (Figure 2G). This distal shift of ray branchpoints suggests that the presence of adjacent intact fin tissue influenced the regenerative program of bone patterning. We concluded that platyfish, like zebrafish, possess a morphogenetic plasticity, regarding the specification of ray bifurcation during fin regeneration.

### Actinotrichia-specific genes, *actinodins*, are co-expressed in the lateral mesenchyme of the regenerative outgrowth

**FIGURE 3:**
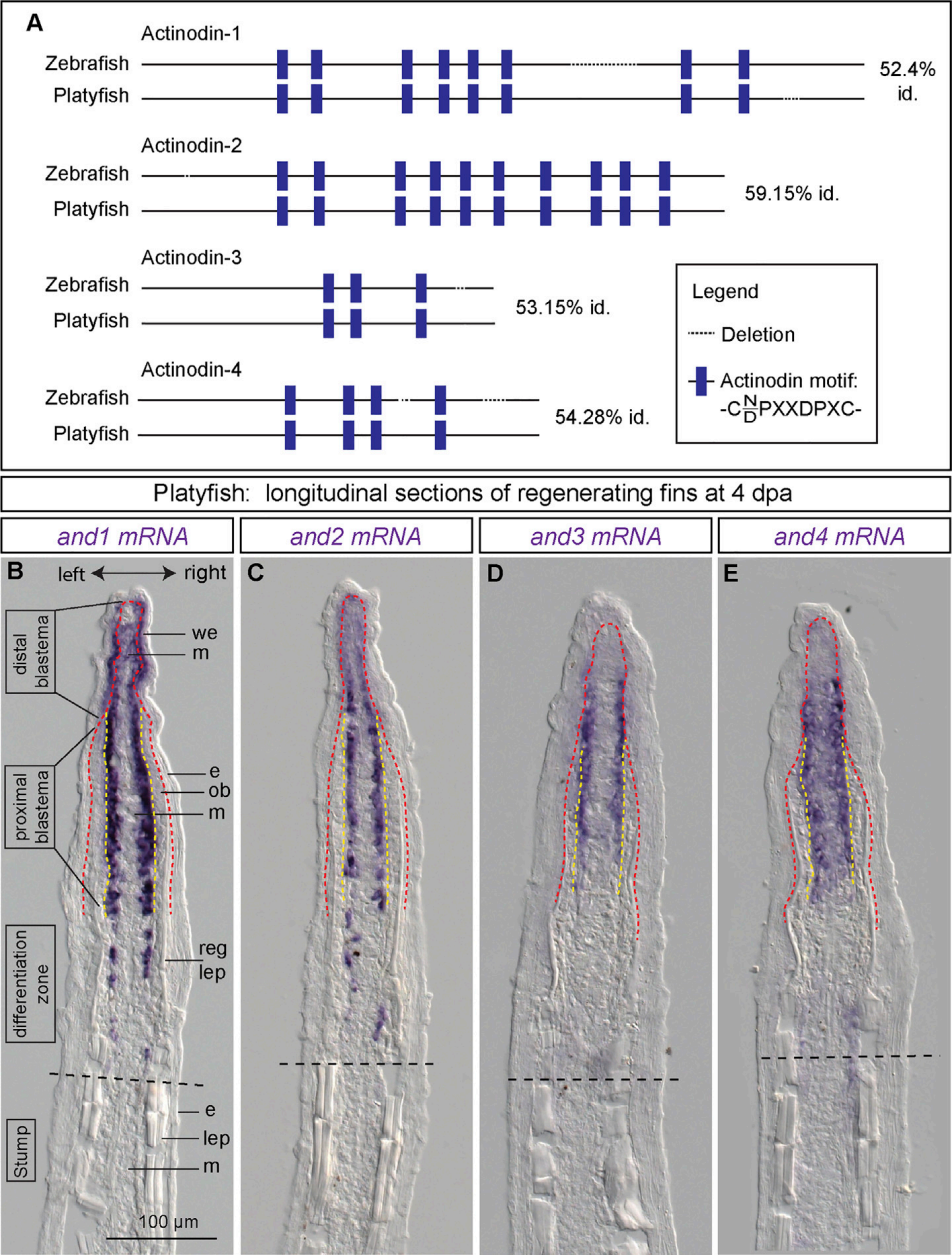
Identification of four *actinodin* genes in platyfish and their expression in the regenerative outgrowth. **(A)** Schematic alignment of *actinodin* orthologs in the zebrafish and the platyfish, identified based on the BLAST tool at NCBI, using the zebrafish annotated proteins as query. Identity (id) represents percentage of the conserved amino acids in the protein sequence. **(B–E)** *In situ* hybridization of *actinodin* genes in the platyfish fin sections at 4 dpa. (N = 3 fish). Black dashed lines indicate amputation planes. Red dashed line depicts the boundary between the wound epidermis and the underlying tissue. Yellow dashed line depicts the boundary between the osteoblast layer and mesenchyme. We, wound epidermis; m, mesenchyme; e, epidermis; ob, osteoblasts; reg lep, regenerating lepidotrichia; lep, lepidotrichia.

The leading tip of regenerated rays is supported by actinotrichia, which serve as the distal-most dermal skeleton of the fin (Figure 1E). These structures comprise specific proteins, called Actinodins, which are recognized by a distinctive peptide motif in their amino acid sequence (Zhang et al., 2010). Using the BLAST tool, we identified platyfish orthologs of the zebrafish *actinodin-1, -2, -3* and -*4* genes (ncbi.nlm.nih.gov). Amino acid alignment revealed an approximately 52%–59% identity between the counterpart proteins, suggesting substantial variations in their primary sequence (Figure 3A). However, the number of the Actinodin peptide motifs was highly conserved between the zebrafish and platyfish homologs. This similar organization of the repetitive motifs suggests a homologous role of the Actinodin proteins in both fish species.

To determine the expression pattern of *actinodin* genes during fin regeneration, we performed *in situ* hybridization of longitudinal sections at 4 dpa. At this time point, three zones of the regenerative outgrowth could be distinguished, based on the presence of the osteoblast layer and the amount of new lepidotrichial matrix, as described in zebrafish (Knopf et al., 2011). Specifically, the distal blastema consists of only the wound epithelium and the apical mesenchyme, whereas the proximal blastema also contains osteoblasts that are positioned between both these tissues. The border between the blastema and the differentiation zone is gradual, corresponding to the maturation of the lepidotrichia. In the distal blastema of the outgrowth, platyfish expressed *and1* in the basal layer of the wound epithelium and in mesenchyme (Figure 3B). In the proximal blastema, *and1* transcripts were no longer detected in the epidermis, but they were exclusively present in the lateral mesenchyme underneath the osteoblast layer. Remarkably, osteoblasts did not display any staining along the entire outgrowth.

Next, we analyzed *and2, and3* and *and4* expression on fin sections. All these probes revealed hybridization-reactivity in the lateral mesenchyme adjacent to osteoblasts (Figures 3C–E). The expression in the distal mesenchyme was weaker than in the proximal blastema. Among these paralogs, *and4* showed additional weak staining in the central mesenchyme of the proximal blastema. None of these paralogs were detected in the wound epidermis and osteoblasts. Taken together, all four *actinodin* genes are co-expressed in the lateral mesenchymal cells underneath the osteoblast layer. In addition, *and1* is detected in the basal wound epithelium. None of the transcripts were observed in lepidotrichia-producing cells (osteoblasts), consistent with the actinotrichia-specific nature of the *actinodin* genes.

### Selective pharmacological inhibition of the BMP type I receptor impairs fin regeneration in platyfish

**FIGURE 4:**
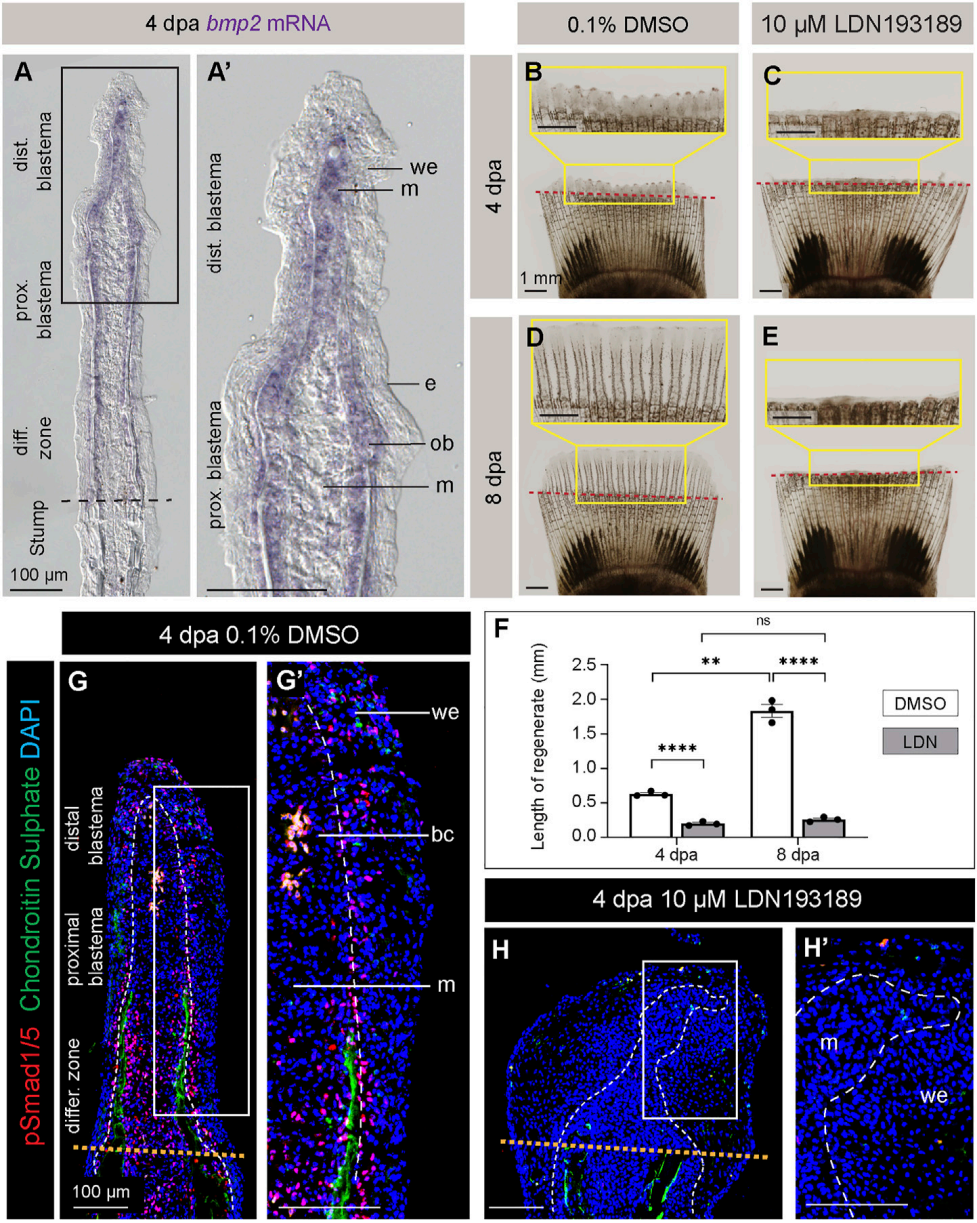
Inhibition of BMP signaling impairs fin regeneration in platyfish. **(A, A′)** *In situ* hybridization of the platyfish *bmp2* gene on fin sections at 4 dpa. **(B–E)** Live imaging of platyfish fins treated with either 0.1% DMSO control or the BMP inhibitor, LDN193189 at 4 and 8 dpa, as indicated on the figure. The regeneration is impaired in the LDN193189-treated group of fish. **(F)** Quantification of the regenerate lengths shows no significant difference between 4 dpa and 8 dpa in the inhibitor-treated group, suggesting a persistent blockage of regeneration, and not merely a delay. N = 3; average of three rays per fin; error bar SEM; n.s., not significant, *p* > 0.05; **p < 0.01; ****p < 0.0001; paired two-tailed Student’s t-test for the same fish at different time-point; unpaired two-tailed Student’s t-test between drug-treated and control fish. **(G and H)** Immunofluorescence staining of fin sections at 4 dpa with antibodies against chondroitin sulphate (green) to visualize lepidotrichia and pSmad1/5 (red), which is a readout of BMP signaling. Orange dotted lines depict the amputation plane; white dashed lines underly the basal epithelium. We; wound epidermis; m; mesenchyme; bc, blood clot. N = 3, at least three ray sections per fin.

Among many molecular signals, the BMP pathway has been shown to play a multifaceted role in restoration of several structural elements of the regenerative outgrowth in the zebrafish fin (Laforest et al., 1998; Quint et al., 2002; Stewart et al., 2014; Thorimbert et al., 2015). The *bmp2b* gene is expressed in regenerating osteoblasts, whereas *bmp2a* is found in the distal blastema. A BLAST search for BMP2 orthologs in the platyfish identified only one amino acid sequence, which is 74% identical to BMP2b and 66% to BMP2a in the zebrafish. To determine whether the *bmp2* gene is expressed in the regenerating platyfish fin, we performed *in situ* hybridization on fin sections at 4 dpa. The staining detected *bmp2* transcripts in mesenchyme of the distal blastema and in osteoblasts of the proximal blastema (Figure 4A, A′). This suggests that BMP signaling might also be involved in fin regeneration in platyfish.

To test the role of BMP signaling in fin regeneration of platyfish, we applied a pharmacological approach. LDN193189 is a selective inhibitor of the BMP type I receptors, called ALK2 (also named ActR-1A and ACVR1) and ALK3 (also named BMPR-1A) (Cuny et al., 2008; Yu et al., 2008). This compound has been functionally validated in the zebrafish fin (Stewart et al., 2014). To inhibit BMP signaling in platyfish, we performed treatment with 10 µM LDN193189, and control fish were incubated with 0.1% DMSO. Live-imaging of fins revealed a strong impairment of regeneration at 4 and 8 dpa (Figures 4B–E). In comparison to control, LDN193189-treated fish had approximately 3- and 7-times shorter regenerative outgrowths at 4 and 8 dpa, respectively (Figure 4F). This result suggests that BMP signaling is required for progression of regeneration in platyfish.

To determine the drug efficacy, we assessed the downstream readout of this signaling by immunostaining against phospho-Smad1/5 (Ser463/465). For this experiment, we fixed fins at 4 dpa and performed longitudinal sections. To distinguish between rays and interrays, we co-labeled the sections with antibodies against chondroitin sulphate, which we identified as a suitable marker of lepidotrichia, like in zebrafish (König et al., 2018). For consistency, we analyzed three sections per fin. In DMSO-treated control specimens, nuclear pSmad1/5 immunostaining was detected in scattered cells in the wound epidermis, in a “salt-and-pepper”-pattern in the blastema, and at a higher density in the region adjacent to the differentiating lepidotrichia (Figure 4G, G′). By contrast, LDN193189-treated fins were shorter than the controls, and they displayed a widened tip (Figure 4H). Indeed, the wound epidermis was thickened and the boundary to the blastema was irregular. No chondroitin sulphate staining was observed distally to the amputation plane, indicating impaired lepidotrichia regeneration. This phenotype was associated with an almost complete absence of pSmad1/5, as we observed only very sporadic immunopositive nuclei in the wound epidermis (Figure 4H′). We concluded that LDN193189 nearly completely suppressed BMP signaling, resulting in impairment of lepidotrichia regeneration and abnormal morphology of the outgrowth.

### Inhibition of BMP signaling reduces cell proliferation and disorganizes the wound epidermis

**FIGURE 5:**
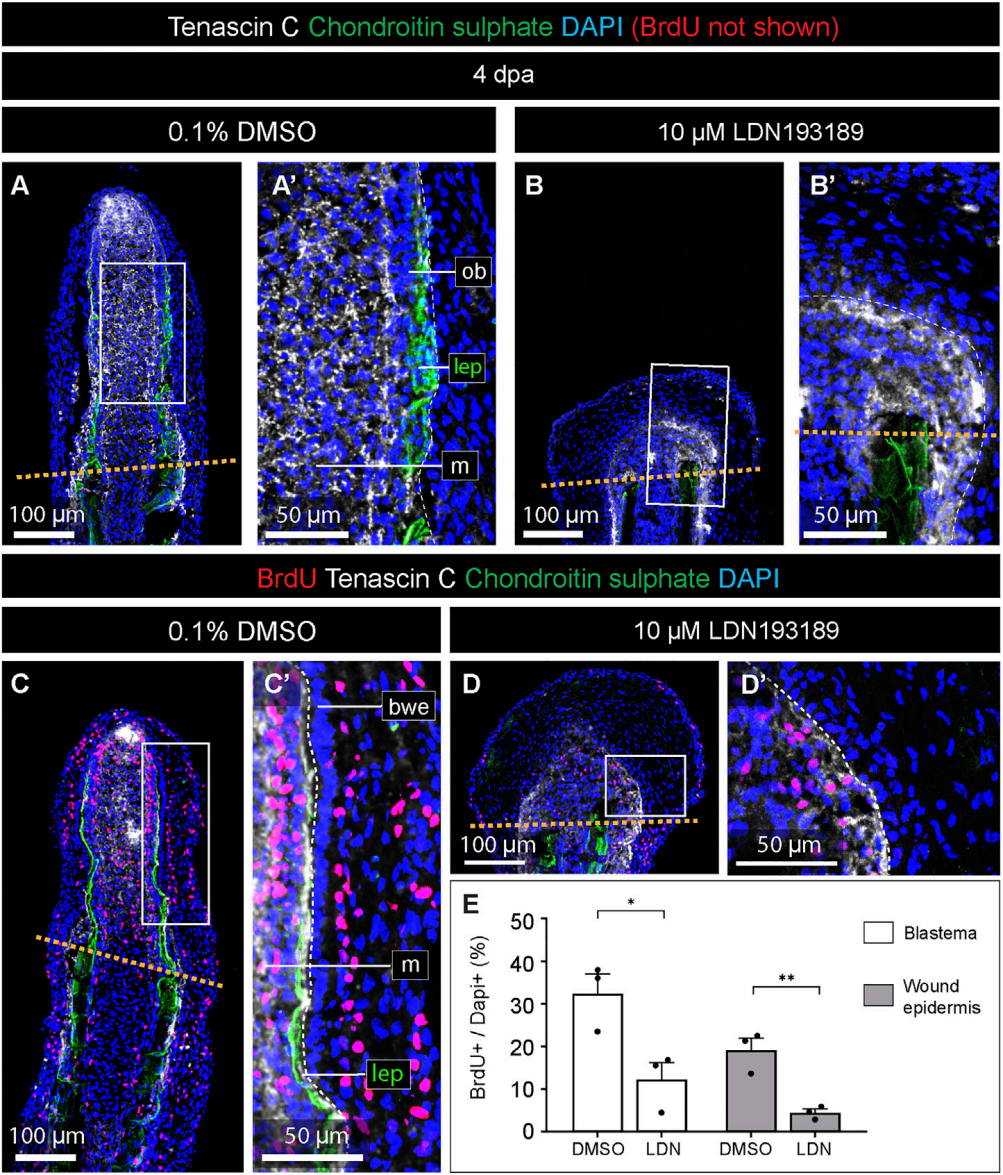
The inhibition of BMP signaling impairs cell proliferation in the blastema and the wound epidermis. **(A–D)** Quadruple fluorescence staining of fin sections at 4 dpa with antibodies against chondroitin sulphate (green) to visualize lepidotrichia, Tenascin C (white) to detect an extracellular protein of the blastema, BrdU (red), and DAPI (blue). Orange dashed lines depicts the amputation plane; white dashed lines underly the basal epithelium (bwe). We; wound epidermis; m; mesenchyme; lep, lepidotrichia. **(E)** Quantification of the proportion of nuclei labeled with BrdU in the two compartments in treated and control fins. N = 3, average of three sections per fin; error bar SEM; *p < 0.05; **p < 0.01; unpaired two-tailed Student’s t-test.

To understand the cellular causes underlying the impaired regeneration of LDN193189-treated fins, we assessed cell proliferation using a BrdU incorporation assay. To label both fast- and slow-cycling cells during outgrowth formation, the fish were incubated with this nucleotide analog for 24 h, a time-window that is at least 3-times longer in comparison to the analogous studies in zebrafish, e.g. (Poleo et al., 2001; Nechiporuk and Keating, 2002; Pfefferli et al., 2014). The incubation started at 3 dpa, and the fins were collected at 4 dpa for immunofluorescence analysis on longitudinal sections. To identify specimens with rays, we co-stained lepidotrichia with the antibody against chondroitin sulphate. To assess tissue remodeling of the blastema, we included an antibody against Tenascin C, which is a matricellular protein, upregulated by Activin/TGFß-signaling during fin regeneration in zebrafish (Jaźwińska et al., 2007). In both control and LDN193189-treated fins, Tenascin C was detected in the mesenchyme of the blastema and underneath the epidermis of the regenerative outgrowth (Figures 5A, B). Thus, in contrast to chondroitin sulphate, Tenascin C was not markedly suppressed by the inhibition of BMP signaling. This observation suggests that BMP signaling is not required for the deposition of Tenascin C and tissue remodeling of the blastema.

Next, we assessed BrdU-positive cells in the Tenascin C-positive blastema and the Tenascin C-negative wound epidermis distally to the amputation plane. In control fins, BrdU-labeling was detected in scattered cells in the blastema and the stratified wound epidermis (Figure 5C). Consistent with previous studies in zebrafish (Poleo et al., 2001; Nechiporuk and Keating, 2002; Thorimbert et al., 2015), the basal epithelial layer did not incorporate BrdU (Figure 5C′). LDN193189-treated fins contained a much smaller proportion of BrdU-immunolabeled cells than control (Figure 5D, D′). Image quantification revealed that the inhibition of BMP signaling resulted in approximately 2.6- and 4.2-fold reduction of the proportion of BrdU-labeled cells in the blastema and wound epidermis, respectively, compared to control (Figure 5E). We concluded that blocking of BMP signaling impairs cell proliferation in both the blastema and the wound epidermis, consistent with the shortened size of the regenerative outgrowth.

**FIGURE 6:**
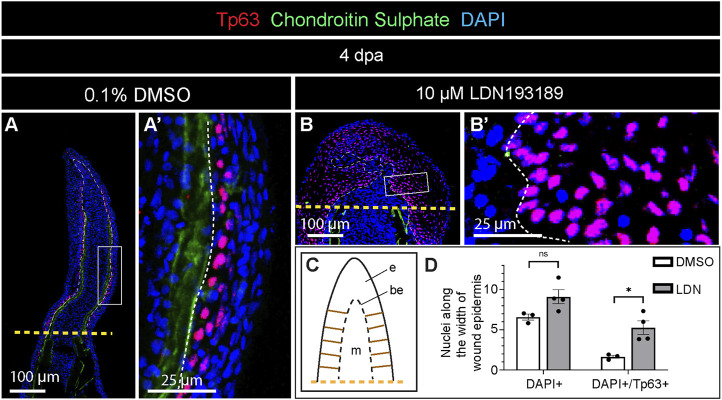
Expansion of Tp63 expression across the layers of wound epidermis in fins treated with the BMP inhibitor during regeneration. **(A and B)** Immunofluorescence staining of fin sections at 4 dpa with antibodies against chondroitin sulphate (green) to visualize lepidotrichia, and Tp63 (red), a marker of the basal epithelium. DAPI (blue) demarcates nuclei. Orange dashed lines show the amputation plane, white dashed lines underly the basal wound epithelium. **(C)** A schematic representation of the fin outgrowth with ladder-like brown lines on each side of the wound epidermis, indicating the approximate positions along which the measurements were performed for quantification of nuclear layers. **(D)** Quantification of nuclei along the width of the wound epidermis as illustrated in **(C)**. N = 3 fins (control) N = 4 (LDN193189-treated fins); three ray sections per fin; error bar SEM; n.s., not significant, *p < 0.05; unpaired two-tailed Student’s t-test.

The thickened wound epidermis represented a conspicuous phenotype in LDN193189-treated fins. To examine the organization of this tissue, we applied an antibody against Tumor protein 63 (Tp63), which is a marker of the basal epidermal layer during homeostasis of the zebrafish fin, but which becomes expressed in suprabasal layers during the healing process (Sousa et al., 2012). At 4 dpa, control fins displayed the nuclear accumulation of Tp63 predominantly in the basal and suprabasal layers of the wound epidermis (Figure 6A, A′). In contrast, LDN193189-treated fins showed extensive Tp63-immunolabelling in the entire stratified epidermis, reaching even the outermost layers (Figure 6B, B′). To quantitatively characterize these changes in the wound epidermis, we counted DAPI- and DAPI/Tp63-stained nuclei along the width of the wound epidermis, which was sampled at 10 interspaced positions (Figure 6C). We found that control fins contained approximately 6 nuclei across the stratified epidermis, and this number was slightly elevated to 9 nuclei in LDN193189-treated fins (Figure 6D). In control fins, Tp63 was expressed in 1-2 epithelial layers, whereas in LDN193189-treated fins, Tp63 expression expanded to five layers (Figure 6D). We concluded that the inhibition of BMP signaling resulted in expansion of the basal layer properties into the more superficial layers of this tissue. Such an abnormal epithelial differentiation might affect the tissue architecture, leading to thickening of the epidermis.

### Inhibition of BMP signaling prevents actinotrichia formation in the blastema

**FIGURE 7:**
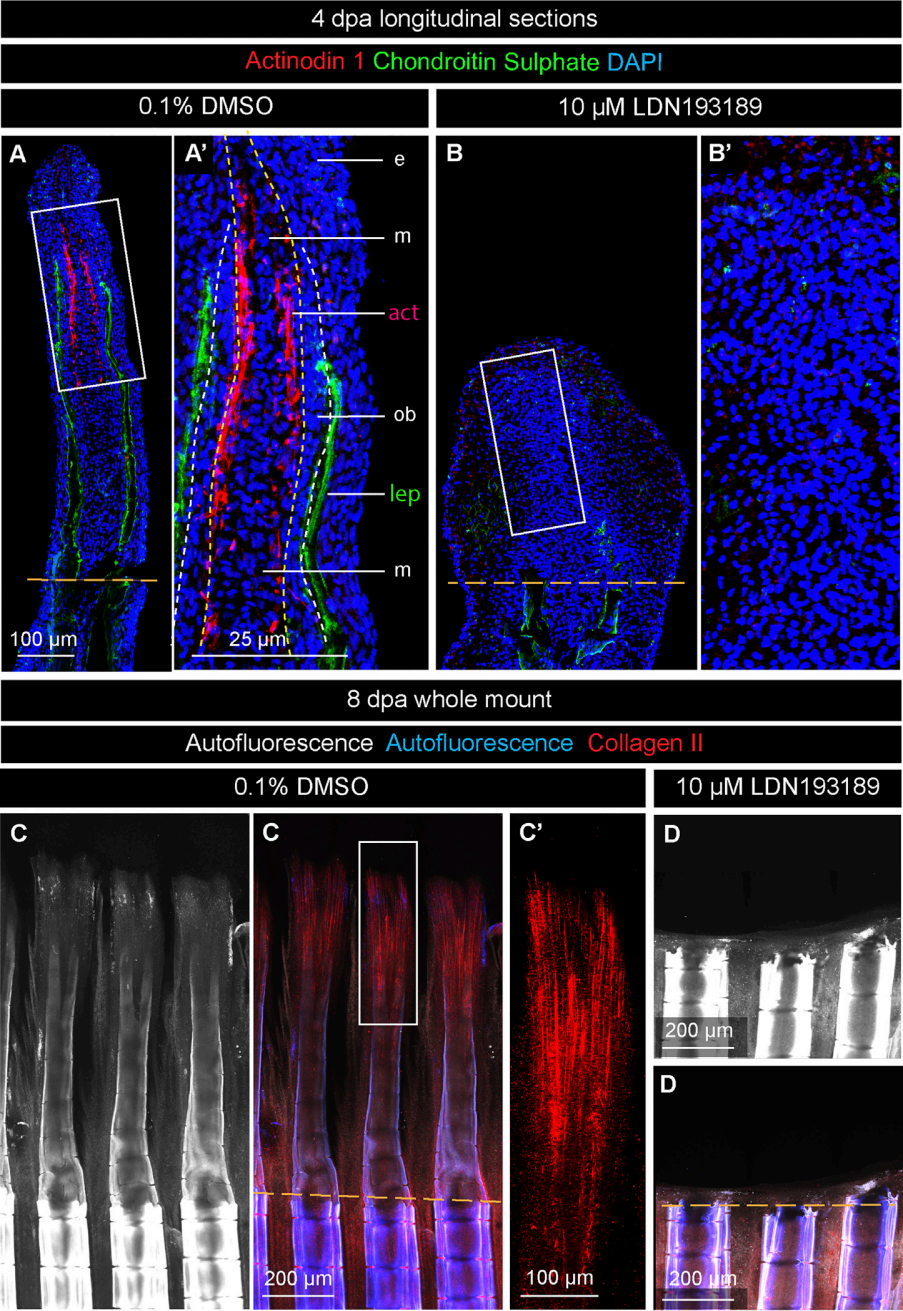
BMP inhibition prevents actinotrichia regeneration. **(A and B)** Immunofluorescence staining of fin sections at 4 dpa with antibodies against chondroitin sulphate (green) to visualize lepidotrichia (lep), and Actinodin 1 (red), a marker of actinotrichia (act). DAPI (blue) demarcates nuclei. Orange dashed lines depict the amputation plane; white dashed lines underly the basal epithelium; yellow dashed line demarcates the boundary between osteoblasts (ob) and mesenchyme (m). e, epidermis. The actinotrichia are located beneath the layer of osteoblasts in control. No Actinodin-1 protein is detected in LDN193189-treated fins. **(C and D)** Fin whole mounts with Collagen II antibody staining (red). The intensity of autofluorescence (white and blue) reflects the level of bone calcification. This parameter indicates the plane of amputation (dashed orange line). (C′) A higher magnification of the framed area in **(C)** displays actinotrichia at the tip of a ray. **(D)** No Collagen II expression is detected in LDN193189-treated fins.

Actinotrichia are essential dermal skeletal elements that support the blastema in the absence of differentiated lepidotrichia. To determine whether BMP signaling is required for actinotrichia formation in platyfish, as shown in zebrafish (König et al., 2018), we generated an antibody against the C-terminal peptide of the platyfish Actinodin-1 (And1) protein. To visualize this actinotrichia-specific protein alongside a lepidotrichia marker, we performed double staining against And1 and chondroitin sulphate on longitudinal fin sections at 4 dpa. In control specimens, the And1 antibody reacted with structures in the lateral mesenchyme in the vicinity of the osteoblast layer of the blastema (Figure 7A, A′). The distribution pattern was reminiscent of the *and1* gene expression pattern, observed in the *in situ* hybridization experiment (Figure 3B). This similarity is consistent with the specificity of our custom-made And1 antibody. In contrast to control, LDN193189-treated fins lacked any And1 staining in the regenerative outgrowth (Figure 7B, B′). This result suggests that the inhibition of BMP signaling impairs deposition of actinotrichia-specific proteins during fin regeneration.

To determine whether Collagen II, which is another component of actinotrichia, is affected by the inhibition of BMP signaling, we analyzed fin whole mounts at 8 dpa. Mature and immature lepidotrichia were distinguished by the intensity of autofluorescence, which was indicative of the amputation plane and the position of the regenerative outgrowth (Figures 7C, D). Control samples displayed an advanced regenerative outgrowth, comprising differentiating rays, whereas LDN193189-treated fins had no markable protrusion beyond the amputation plane (Figures 7C, D). Only in control fins, Collagen II was detected in actinotrichia, which formed brush-like structures at the tip of regenerating rays (Figure 7C, C′). A lack of Actinodin-1 and Collagen II staining in LDN193189-treated specimens suggests that BMP signaling is required for actinotrichia formation during fin regeneration in platyfish.

## Discussion

**FIGURE 8:**
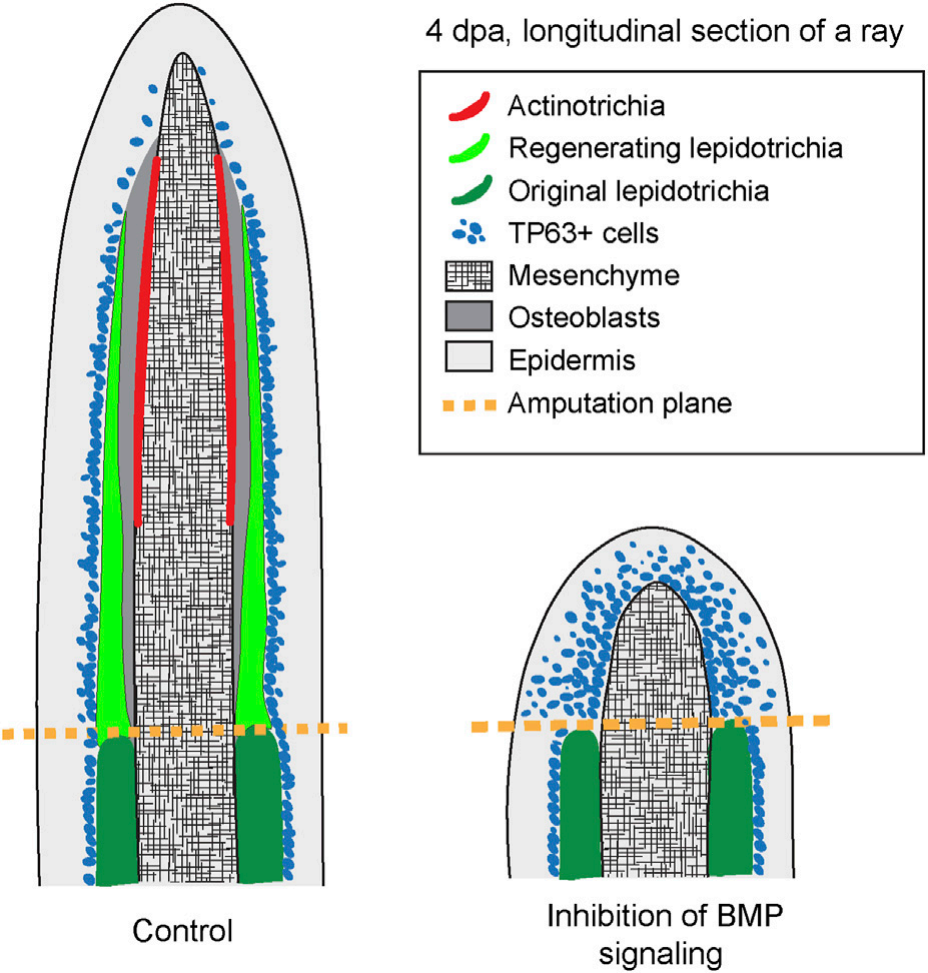
A schematic summary of the phenotype caused by the inhibition of BMP signaling during outgrowth formation following fin amputation in platyfish. The schematic is based on immunofluorescence staining of fin sections at 4 dpa. Pharmacological inhibition of BMP signaling prevents regeneration of actinotrichia and lepidotrichia. Furthermore, the nuclear marker of the basal epidermal layer, Tp63, is abnormally detected throughout all layers of the wound epidermis in LDN193189-treated fins, suggesting an abnormality in the differentiation of this tissue.

Caudal fin regeneration after partial amputation represents a common capacity of bony fishes. This process has been extensively studied in the zebrafish and the medaka, thanks to an advanced repertoire of genetic tools for these model organisms. Many other species have been reported to regenerate their fins, including the *Xiphophorus* taxon, represented by the swordtail (*Xiphophorus hellerii*) and the platyfish (*X. maculatus*) (Offen et al., 2008). To expand the understanding of fin restoration in platyfish, we focused our study on morphogenetic ray plasticity, dermal skeleton regeneration and wound epidermis differentiation, which remain poorly explored in the advanced orders of neoteleosts. Firstly, we found that platyfish fin regeneration can occur without a distal shift of ray branchpoints, which is typical for the zebrafish fin. Secondly, the analysis of *actinodin* genes revealed that all four members of this protein family are expressed in the lateral mesenchyme of the regenerative outgrowth, suggesting their contribution in actinotrichia formation. Thirdly, the pharmacological inhibition of BMP signaling brings additional insights about the role of this pathway in stimulation of blastemal cell proliferation, dermal skeleton deposition, and the compartmentalization with the wound epithelium (Figure 8). Taken together, our study expands our knowledge about appendage restoration in neoteleosts and provides information for the identification of relevant similarities and differences between phylogenetically remote clades of bony fish.

In zebrafish, an amputated caudal fin can restore its size and shape. However, ray branchpoints are not reproduced according to the original pattern, but they are shifted distally (Akimenko et al., 2003; Azevedo et al., 2011). This imperfection remains a mystery, despite the availability of powerful genetic approaches for this species. Interestingly, we found that this phenomenon is not a default program in platyfish. We do not yet understand why platyfish, in comparison to zebrafish, can more precisely reproduce the original position of the bifurcation points. One explanation for this difference could be related to the shape of the appendage. While the zebrafish caudal fin has a concave margin (Bird and Mabee, 2003), the platyfish counterpart has a convex geometry (Figures 1A, 2A). Whether this inverse architecture might affect hydrodynamic properties that regulate ray bifurcation requires further interdisciplinary studies. Indeed, our collaborative projects have previously suggested that fluid dynamics might provide cues for the ray-interray distribution of sensory cells and for the position of ray bifurcations in the regenerating zebrafish fin (Puri et al., 2017; König et al., 2019; Dagenais et al., 2021). In agreement with the hypothesis of the extrinsic input for ray morphogenesis, our experiments with ray excision triggered a distal shift of ray branchpoints. Thus, the interaction between remaining tissue and newly forming rays seems to play a non-autonomous role in ray patterning in platyfish. In summary, although the position of primary ray bifurcations can be faithfully reproduced after straight amputation of the platyfish fin, the rays possess the intrinsic capacity to adapt to variations of the appendage shape.

Fin regeneration depends on a coordinated network of signaling pathways that organize wound healing, cell dedifferentiation, proliferation and redifferentiation of the tissue, until the original size has been reached (Wehner and Weidinger, 2015). One of the signaling pathways implicated in this process is BMP, which regulates osteoblast maturation, actinotrichia formation, mesenchymal cell proliferation, blood vessel morphogenesis, and wound epithelium differentiation in the regenerating zebrafish fin (Smith et al., 2006; Stewart et al., 2014; Wehner et al., 2014; Thorimbert et al., 2015). Here, we report that pharmacological inhibition of BMP type 1 receptors led to several similar abnormalities in platyfish. This suggests a conserved role of this pathway in fin regeneration among teleosts.

To expand our understanding of the function of BMP signaling in the wound epidermis, we investigated the localization of the Tp63 protein. We focused on this marker because this transcriptional factor is essential for epidermis development and differentiation in mammals (Botchkarev and Flores, 2014; Soares and Zhou, 2018). Its main role is to keep basal epidermal layer cells in quiescence and to protect them from premature depletion (Su et al., 2009). Consistently, we identified that Tp63 is expressed in the quiescent basal layer of the wound epidermis in the regenerative outgrowth of the platyfish fin. Interestingly, blocking BMP signaling broadened Tp63 expression throughout the superficial epidermal layers. The expansion of Tp63 expression was associated with a reduction of cell proliferative activity, compared to control fins. Thus, our findings are in line with the function of Tp63 in mammals. Accordingly, the interpretation of the thickened appearance of the wound epidermis might involve several aspects, including impaired differentiation of the squamous layers or tissue inflation at the level of extracellular matrix. Further studies are required to determine specific Tp63-dependent mechanisms.

Studies of zebrafish embryos have demonstrated that an N-terminally truncated isoform of Tp63 is needed for epidermal growth and fin development, and this transcription factor is a direct target of BMP signaling (Bakkers et al., 2002; Lee and Kimelman, 2002). The regulation of Tp63 by BMP is also observed in our experiments in the platyfish fin. Specifically, our data indicated that Tp63 is negatively regulated by BMP signaling in the suprabasal epidermal layers of the regenerative outgrowth. It would be interesting to determine whether Tp63 is a BMP target in the regenerating caudal fin in zebrafish.

Fin regeneration relies on lepidotrichia and actinotrichia formation. Actinotrichia are the first dermal skeleton structures that are established to support the immature blastemal outgrowth. We showed that platyfish require BMP signaling to restore actinotrichia and lepidotrichia. Similar results were reported in zebrafish experiments with pharmacological approaches (Stewart et al., 2014; Thorimbert et al., 2015). In the zebrafish fin, BMP signaling is also involved in promoting blastemal cell proliferation, as indicated by overexpression of the inhibitor Chordin (Smith et al., 2006). It remains to be determined whether impaired proliferation in the mesenchyme represents a primary or secondary effect caused by the lack of dermal skeleton in the blastema.

In conclusion, our study revealed several new aspects of caudal fin regeneration in platyfish. Identification of specific environmental and geometrical cues that control ray branching plasticity would require additional interdisciplinary approaches to be fully understood. Further studies are necessary to dissect the molecular basis underlying ray morphogenesis and patterning. The analysis of the phenotype caused by the inhibition of BMP signaling supports the idea that this pathway orchestrates regeneration of multiple tissues of the complex appendage in different fishes. In platyfish, BMP is required for organization of the layers in the wound epidermis, cell proliferation in the regenerative outgrowth and for lepidotrichia and actinotrichia formation. All these functions are fundamental for the progression of fin regeneration, and they seem to be conserved between phylogenetically remote groups of bony fishes, namely *Otophysa* and neoteleosts.

## Data Availability

The original contributions presented in the study are included in the article/Supplementary Material, further inquiries can be directed to the corresponding author.
