# Supplementary figures and images for: Regeneration of the dermal skeleton and wound epidermis formation depend on BMP signaling in the caudal fin of platyfish
Source: Front Cell Dev Biol. 2023 Feb 9;11:1134451. doi: 10.3389/fcell.2023.1134451 (PMC9946992; doi:10.3389/fcell.2023.1134451)

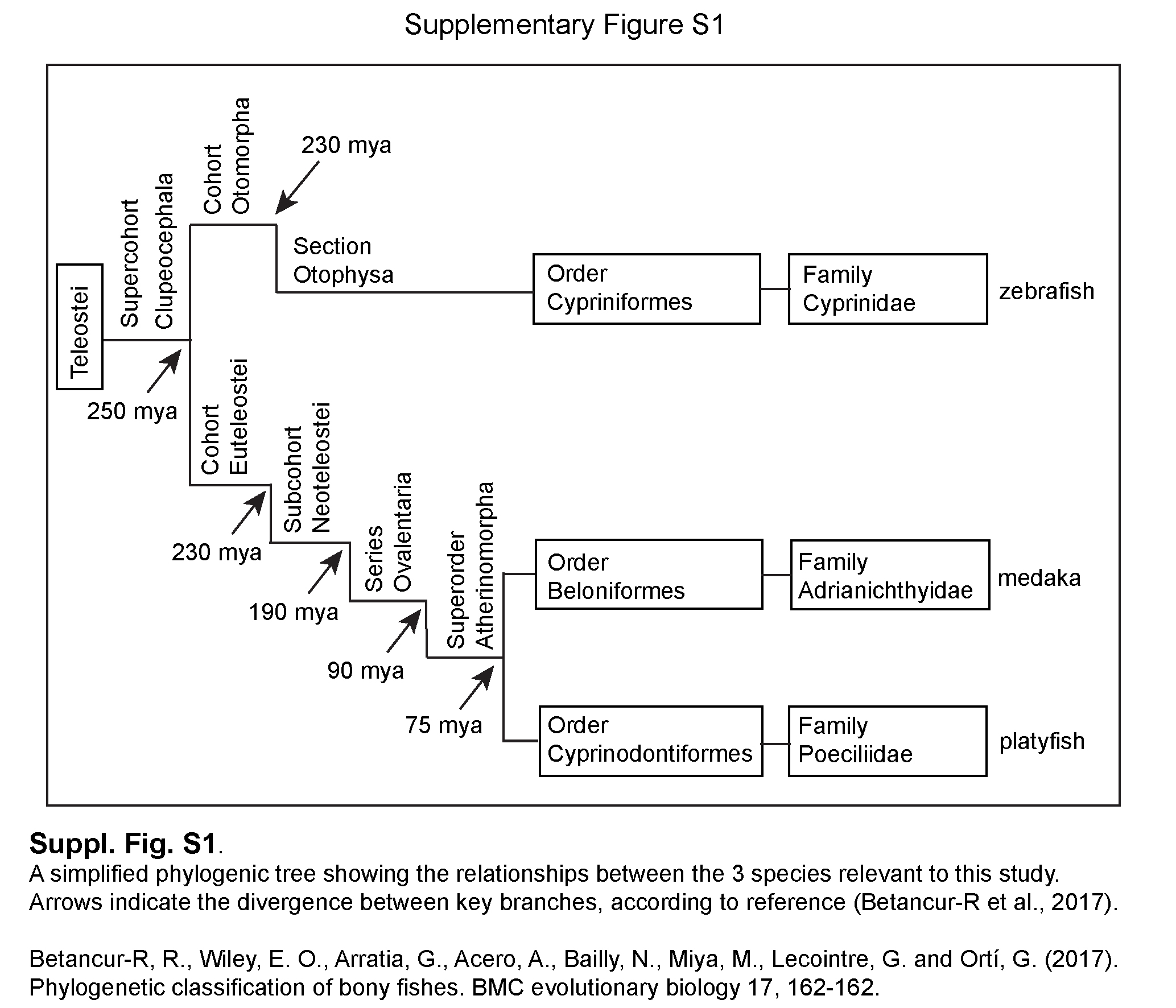

Supplement: Supplementary file 1 [file Image1.JPEG]
